# Next-generation CRISPR/Cas-based ultrasensitive diagnostic tools: current progress and prospects

**DOI:** 10.1039/d4ra04838e

**Published:** 2024-10-14

**Authors:** Deepak Kumar Sahel, Gangadari Giriprasad, Reena Jatyan, Sonia Guha, Aishwarya Korde, Anupama Mittal, Sunil Bhand, Deepak Chitkara

**Affiliations:** a Department of Pharmacy, Birla Institute of Technology and Science, Pilani Vidya Vihar Pilani 333031 Rajasthan India deepak.chitkara@pilani.bits-pilani.ac.in +91 01596 515 835 +91 9660 456 009; b Department of Chemistry, KK Birla Goa Campus, BITS Pilani Goa 403726 India

## Abstract

CRISPR/Cas has been explored as a powerful molecular scissor that uses a double-strand break mediated non-homologous end joining (NHEJ) or homology-directed repair (HDR) to achieve precise gene editing. Cas effectors come in several different forms, each with its own set of features and applications. SpCas9 was the first and most extensively studied CRISPR/Cas version, and it has been hailed as a biotechnology breakthrough that could potentially correct mutations to treat genetic diseases. Recently, the Cas12 and Cas13 effector variants of Class II, Type V and Type VI, have been explored for their specific collateral cleavage (*trans*-cleavage) activity on target recognition. This *trans*-cleavage activity helps in the recognition of target nucleic acids. CRISPR diagnostics technology utilized the binding of crRNA with Cas12/13 protein to form the Ribonucleoproteins (RNPs) complex, which further cleaves the target sequence in *cis*-cleavage, followed by the activation of *trans*-cleavage of a nonspecific fluorescent DNA/RNA probe, resulting in the production of a fluorescent signal that could be quantitatively recorded. Later, nanotechnology and mobile-based detection applications were incorporated into the system to develop advanced lateral flow-based strips and are also associated with the technology to make it more feasible. Overall, this review compiles the experimental evidence consolidating the application of CRISPR/Cas as next-generation biosensors for diagnostic applications.

## Abstract 2

CRISPR/Cas has been explored as a powerful molecular scissor that uses a double-strand break mediated non-homologous end joining (NHEJ) or homology-directed repair (HDR) to achieve precise gene editing.

## Graphical abstract

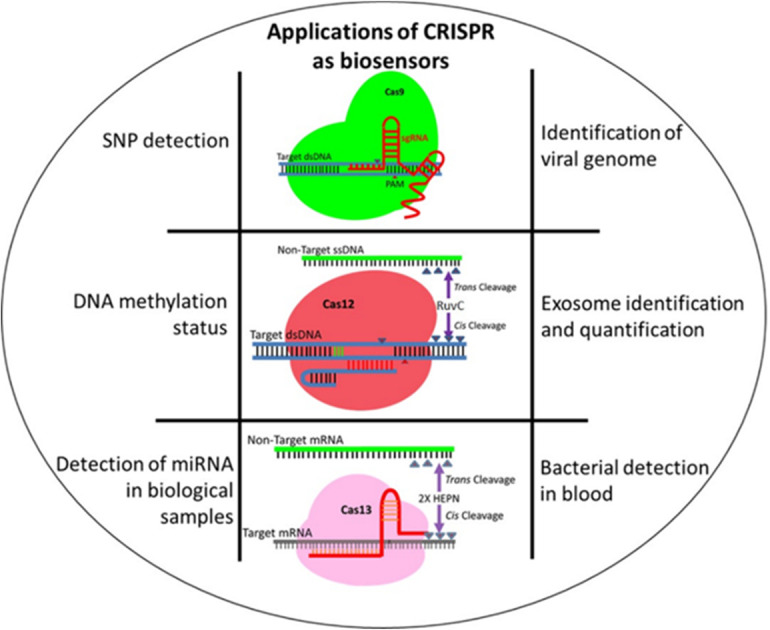

## Introduction

Clustered regularly interspaced short palindromic repeats (CRISPR) is a well-known emerging gene-editing tool hailed as a breakthrough in the field of biotechnology because of its precise and specified gene editing efficiency.^1,2^ The tool is being utilized for the treatment of various genetic and non-genetic diseases utilizing its double-strand break (DSB) mediated DNA repair activation system. The heart of the system lies within a CRISPR-associated protein named Cas9, having endonuclease property directed by a 100-nucleotide long single guide RNA (sgRNA).^3^ The sgRNA comprises a tracrRNA (common for a single Cas9 variant) and a crRNA (that could be switched and having complementarity to the target sequence). CRISPR can be used in many forms such as plasmid (having Cas9 and sgRNA insert), mRNA (which translates downstream to form Cas9), and pre-assembled Cas/sgRNA ribonucleoprotein (RNP) complex.^4^ The RNP is the foremost form of CRISPR with ample advantages such as time effectiveness, less off-target effect, less mutagenesis, *etc.*, over existing forms.^5^ Two classes, six types, and 33 subtypes of CRISPR have been discovered,^6,7^ where all of them have distinct properties. Despite DSB, CRISPR has been explored for its applications such as gene regulation, epigenome modification,^8^ diagnosis,^9^ and theranostic.^10^ Numerous diseases such as cancers, diabetic complications, and neurodegenerative diseases become severe due to a lack of early-stage diagnosis, culminating in uncontrolled mortality. More specifically, people with diabetes mellitus encounter death due to complications such as retinopathy, nephropathy, cardiomyopathy, *etc.*^11^ Likewise, in cancer, early-stage diagnosis is the need of the hour.^12^ Gootenberg *et al.* showed that CRISPR could identify the EGFR L858R mutation or exon 19 deletion in patients with non-small cell lung cancer (NSCLC).^13^ Similarly, CRISPR has the potential to detect gene mutations in cancer patients,^14^ which confirms that the cancer therapies and diagnostics will surely be transformed by CRISPR-Cas enzymes, resulting in clinical benefit. The pandemic caused by COVID-19 infection has generated enormous interest in viral infection detection and led to the development of a powerful technology for infection diagnosis by detecting the viral genome in biological samples. CRISPR-based diagnostics, which make use of its site-specific binding/cleavage capability, are the most significant, versatile, and widely adopted technology being developed.^9^ For many years, nucleic acids have been major markers for the detection of disease progression, and there are many nucleic acid detection assays approved for clinical use. The existing nucleic acid detection assays/methods have limitations such as time consumption, cost, non-reliability, less specificity and precision.^15^ In this review, we looked at the experimental evidence for CRISPR biosensors and their potential for the early identification of lethal diseases *via* nucleic acid detection. We attempted to discuss the advantages of the existing diagnostic techniques over the past and their challenges, as well as future perspectives of amplification-free CRISPR/Cas techniques. We tried to highlight the challenges of the emerging techniques that will contribute to developing more efficient strategies in biosensing and hold tremendous promise to become the next generation of detection techniques. Furthermore, we have shed light on the role of nanotechnology in the long-term use, in terms of the stability and adaptability, of the aforementioned CRISPR diagnostics.

## Biosensors

Detection of biomolecules in a biological sample could provide information about the history and imminence of any possible or existing complication. Therefore, the term biosensor, which means something to detect and analyze any biomolecule, comes into existence.^16^ Biosensors are designed with specificity and accuracy for their target in any biological sample such as serum, urine, plasma, blood, cell extract, *etc.* A biosensor device might be made up of a variety of components competent in identification, transduction, and signal processing. Hence, potentially providing speed, real-time measurement, ease of use, and accuracy in molecular analysis.^17^ In recent times, biosensors have interestingly provided ample therapeutic applications in the detection and early-stage diagnosis of various diseases.^18^ In the development of biosensors, biochemical or molecular markers play an important role. For example, troponin and myoglobin are used as cardiac biomarkers in serum which are detected by a biosensor. Insulin autoantibodies (IAA) and antibodies to the tyrosine phosphatase-like protein IA-2 are diabetes-related autoantibodies. Surface plasmon resonance (SPR) biosensor assays were designed for both autoantibodies.^19^ An “indirect competitive immunoassay” employing biosensors with immobilized insulin is used to detect IAA. Similarly, many biomarkers have been found and investigated for use in diagnosis, as reported elsewhere.^20,21^

## Nucleic acid as a biomarker in diagnosis

Nucleic acid detection is a critical approach in the diagnostic sector, and numerous diagnosis assays that use existing technologies, such as synthetic biomolecular components, polymerase chain reaction (PCR), and fluorescent probes, have been created for the rapid detection of the same,^22^ but all these methods do not meet satisfactory outcomes due to a lack of sensitivity,^23^ specificity, simplicity, cost-effectiveness, and speed of assay.^24^ Urdea *et al.* published an article advocating for the development of a fresh high-impact diagnostic tool to detect an emerging disease at an early stage.^25^ Biosensors utilizing nucleic acids as the recognition component are categorized as affinity sensors. The sensor detects the binding interaction between the nucleic acid and the analyte, as well as the accompanying physicochemical changes, allowing nucleic acid-based biosensors to be employed to detect a wide range of targets such as bacteria and viruses.^26^ The establishment of biosensors for the detection of nucleic acids offers several benefits for biochemical research,^27^ medical diagnosis,^28^ and therapeutic applications.^29^ For instance, Guo *et al.* reported a nanosensor based upon surface-enhanced Raman spectroscopy (SERS) for detecting nucleic acids that are directly correlated with the onset of diseases such as prostate cancer. Therefore, the method could aid in a better therapeutic approach by assisting physicians with treatment options.^29^ Another example is presented by Wang *et al.*, wherein a catalytic hairpin assembly-based (CHA) electrochemical biosensor was developed for the detection of MXR7 gene, which is a marker of liver cancer.^28^ Additionally, Xu *et al.* utilized loop-mediated isothermal amplification (LAMP) to develop a nucleic acid biosensor to identify adulteration in meat on-site.^27^ Biosensors for detecting nucleic acids usually rely on the hybridization of nucleotide probes and target sequences for identification by several transduction mechanisms, such as optical, electrochemical, and mass biosensors.^30^

## CRISPR/Cas systems

**Fig. 1:**
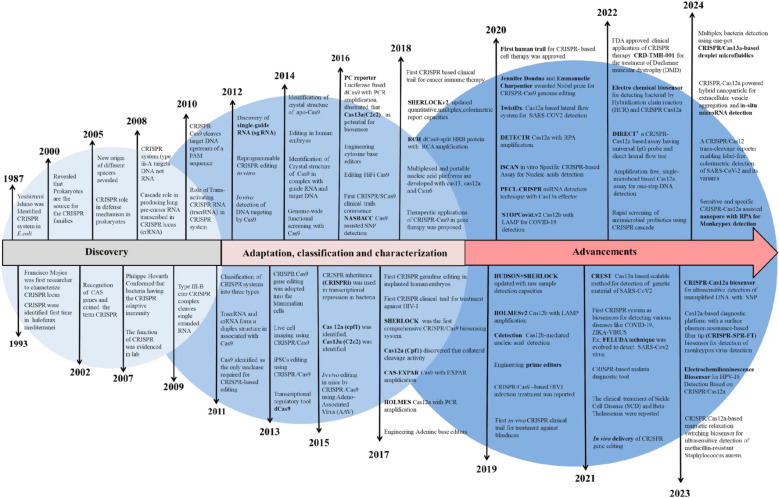
Evolutionary timeline of the clustered regularly interspaced short palindromic repeats (CRISPR).

CRISPR was initially found as a virus-fighting adaptive immune system in bacteria and archaea, where it functions in tandem with Cas9, a CRISPR-associated endonuclease protein.^31^ Later, its mechanistic approach revealed that the CRISPR immune response is caused by three stages: adaptation, expression, and interference.^32^ Cas9 recognizes a specific site on DNA called the protospacer adjacent motif (PAM) during adaptation, then cleaves the DNA.^33^ Following that, the cleaved region is reverse-transcribed at the 5′ end before being inserted into the array as a spacer. During expression, a single transcript, *i.e.*, pre-CRISPR RNA (pre-crRNA), is transcribed by a CRISPR array, which is later processed to form a mature CRISPR RNA (crRNA).^34^ The obtained crRNA has the same sequence as that of the spacer. The maturation of pre-crRNA to crRNA is controlled by various single or multiple Cas domain or non-Cas host RNases. During interference, the crRNA acts as a guide to identifying the sequence in the upcoming viral genome, and after identification, the Cas protein inactivates it. At every stage of the CRISPR/Cas mechanism, various effector proteins and downstream components work together to in-line the process. Like other bacterial systems, the CRISPR/Cas system also has diversity in terms of the sequence of Cas protein, composition, and architecture of genomic loci. The knowledge of CRISPR/Cas diversity keeps growing, and according to the latest classification by Makarova *et al.*, CRISPR/Cas was discovered with two classes, six types, and 33 subtypes.^6^ The most explored CRISPR is type II, which originated from *Streptococcus pyogenes* (spCas9).^35^ In the past decade, the number and the diversity of CRISPR/Cas variants have increased and therefore have been adopted and explored as a tool with ample applications such as epigenome editing, RNA editing, gene suppression/expression (CRISPRi/a), diagnostics, *etc.* A detailed timeline of the CRISPR evolution is shown in Fig. 1.

## CRISPR/Cas in nucleic acid diagnostics

**Fig. 2:**
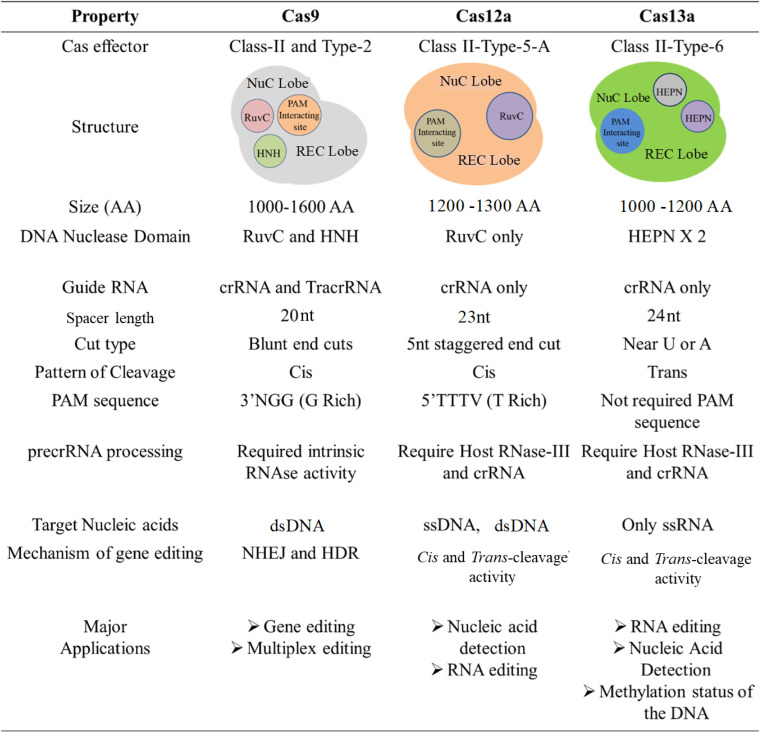
Physiochemical properties of different Cas effector proteins.

Interestingly, the repurposing of the CRISPR target specificity in diagnostics caught the limelight as it could fulfill the translational necessities to the early-stage diagnosis of various diseases. In recent advances, CRISPR/Cas system class II, especially type V, which encodes Cas12a, also called Cpf1 protein, has come under the spotlight for its unique nature. It has been stated that Cas12a has intrinsic RNase activity and does not require tracrRNA/RNaseIII in the maturation of its crRNA. Cas12a itself converts pre-crRNA to mature crRNA and therefore acts as a unique effector protein with endonuclease and endoribonuclease properties.^36,37^ Like Cas9 effector protein, Cas12a also has a RuvC-Nuc domain but does not have an HNH-Nuc domain, thus having a single nuclease site (*i.e.*, RuvC).^37^ Unlike Cas9, which results in blunt ends after the double-strand break (DSB), Cas12a leads to the formation of staggered ends in the genome after cleavage.^38^ Further, Cas12a/b effector proteins have been recently explored for their precise nucleic acid detection-based diagnosis application.^39^Fig. 2 shows an overview of the properties of different Cas effectors.

## Workflow of CRISPR-based biosensors

**Fig. 3:**
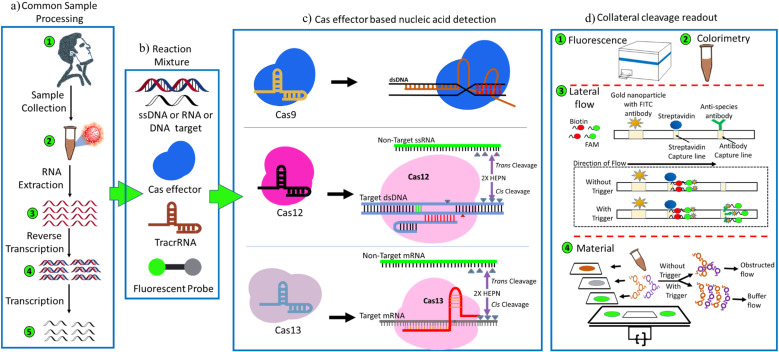
Overview of the basic mechanism involved while adopting CRISPR as biosensors, starting from (a) sample processing, (b) common reaction pool, (c) Cas effector-based *cis*–*trans* cleavage, and (d) post-cleavage detection.

The biosensing principle of the CRISPR-based biosensors mainly depends upon two mechanisms, namely (a) cleavage-based and (b) binding-based.^40^ Both these mechanisms involve three steps: (i) signal amplification, (ii) signal transduction, and (iii) signal reporting. In binding-based CRISPR/Cas biosensing systems, Cas effector proteins can bind the target nucleic acids for their specific detection. These effector proteins are combined with a split enzyme or split fluorescent protein. Enzymatic activity of the Cas effector protein is initiated, generating signals for easy detection.^41^ Nuclease-deactivated Cas9 (dCas9) effector protein was primarily used in this method, and FELUDA is an example of the same. The binding of dCas9 to a target nucleic acid brings re-insertion of the cleaved fluorescent protein or enzyme, producing the detection signals.^42^ Examples include a paired dCas9 (PC) reporter system^43^ and an RCH technique^44^ (rolling circle amplification, CRISPR-Cas9, and split-horseradish peroxidase). On the other hand, the cleavage-based CRISPR/Cas biosensing methods utilize Cas effector proteins that have collateral activity on target nucleic acids. These effector proteins form a ternary complex, crRNA (or sgRNA), target nucleic acid, and the collateral ssDNA or ssRNA reporter undergoes *trans*-cleavage activity to create small fragments.^45^ This collateral cleavage activity is triggered by the ternary complex and targets nucleic acid. Cas9, Cas12, and Cas13 effector proteins were employed in this method.^46^ Cas12 effector creates a ternary complex with the targeted nucleic acid and *trans*-cleaved the collateral ssDNA reporter to tiny pieces in the presence of sgRNA. In parallel, Cas13 effector protein has collateral cleavage activity on ssRNA and target RNA under the supervision of sgRNA. The ssDNA (or ssRNA) reporter could be marked with fluorescence and quencher or biotin-FAM, and detachment of the reporter can be easily observed using a fluorescence reader or colorimetric change in a paper lateral flow experiment.^47^ A brief overview of the basic methodology of CRISPR-based diagnosis is shown in Fig. 3. Examples of this strategy include iSCAN-V2, SHINE, HUDSON + SHERLOCK, STOP-Covid, APC-Cas, HOLMESv2, CAS-EXPAR, DETECTR, NASBACC, and SHERLOCKv2.

## CRISPR-based biosensors: extensive evidence

**Fig. 4:**
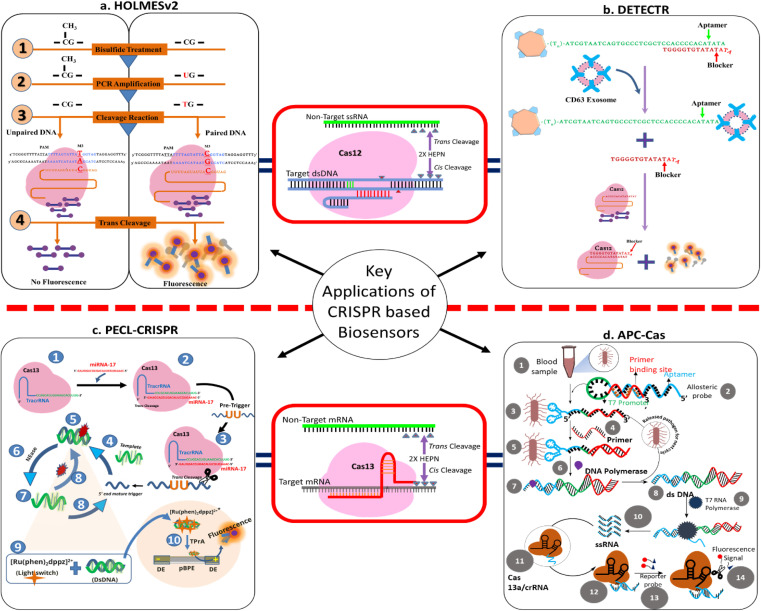
Schematic illustration of key applications of CRISPR-based biosensors. (a) HOLMESv2: detection of DNA methylation status. (b) DETECTR: detection of exosomes. (c) PECL-CRISPR: detection of micro-RNA. (D) APC-Cas: detection of pathogens (*e.g.*, bacteria) in biological samples.

Several CRISPR-based diagnostics have been reported that utilize either the cleavage-based or the binding-based strategy for the detection of nucleic acids (Table 1). The area is constantly evolving, and CRISPR-based diagnostics have shown promise as a mainstream platform for next-generation biosensing. Firstly, the Cas9 effector was explored to distinguish SNPs and provide single base resolution. To conclude, this technique used the Cas9 effector's cleavage activity to cleave the nucleic acid sequence-based amplified (NASBA) Zika virus DNA. The cleavage activity of Cas9 was restricted to the NASBA Zika virus DNA owing to specially designed sgRNA. Nucleic acid sequence-based amplification-CRISPR cleavage (NASBACC) was the name given to this technology and is an example of cleavage-based CRISPR diagnosis.^71^ The catalytically inactivated effector Cas9 protein, *i.e.*, dCas9, provides binding-based diagnosis, in which the split protein/enzyme fused dCas9 binds to nucleic acid without causing any cleavage, allowing the split protein/enzyme to re-integrate, resulting in a fluorescence signal. On the other hand, the discovery of collateral RNA or ssDNA cleavage properties of Cas effector proteins, specifically Cas12 and Cas13, paves the way to developing CRISPR diagnostics that can detect nucleic acids along with ample downstream applications (Fig. 4). When Cas12 binds to crRNA and nucleic acid, it forms a ternary complex, which enables the ssDNA reporter to be collaterally cleaved. Likewise, Cas13 exhibits collateral cleavage of ssRNA reporter. The general premise lies in the technology of the fluorescent and quencher, or any other ligand, such as biotin-FAM, that can be tagged to these ssRNA or ssDNA reporters, and the dissociation of the reporter produces a fluorescent or colorimetric signal, which can then be measured *via* analytical techniques. Signal amplification, signal transduction, and signal reporting were the three essential stages in CRISPR-based biosensing. Here, we explore some recently reported CRISPR diagnostics.

**Table 1:** Different nucleic acid detection methods using CRISPR/Cas effector proteins

No.	Diagnostic tool	Mechanism	Cas variant	Target disease	Fluorescent probe	Collateral readout method	Sensitivity	Outcomes	Time/cost	Advantages	Reference
1	CRISPRD	Utilized *trans*-cleavage activity of Cas12b and Cas13a	*Alicyclobacillus acidiphilus* (AapCas12b), *Thermoclostridium caenicola* (TccCas13a)	High-risk human papillomavirus (hrHPV) detection	ssDNA-labelled ROX reporter, ssRNA-labelled HEX reporter, ssRNA-labelled FAM reporter	Fluorescence	CRISPRD having limit of detection of 10 copies per μL and 100% specificity	CRISPRD enabled the first highly specific and sensitive triplex hrHPV detection method, a step toward decentralizing hrHPV diagnostics	<1 h	It can be adapted for the multiplex detection of any two nucleic acid biomarkers	^48^
2	RPA-CRISPR-Cas12a	Utilized *trans*-cleavage activity of Cas12a	*Lachnospiraceae bacterium* (Lba Cas12a)	Fuso bacterium nucleatum (Fn) detection	ssDNA-labelled FAM reporter	Fluorescence and lateral flow assay	Sensitivity validation revealed a limit of detection of 5 copies per μL	Rapid and sensitive method was developed using CRISPR-Cas12a for the detection of Fn	30–40 min	It has the potential to play an increasingly important role in infectious disease testing	49
3	CRISPR/Cas12a system with DMF	Utilized *trans*-cleavage activity of Cas12a	*Lachnospiraceae bacterium* (Lba Cas12a)	*Staphylococcus aureus* detection	ssDNA-labelled FAM reporter	Fluorescence and digital microfluidic chip	The detection limit of the assay was found to be 32 CFU mL^−1^	DMF-Cas12a method shows good potential in automated and highly sensitive pathogen detection	55 min	This platform was successfully applied to urine and milk samples with excellent sensitivity and reliability	50
4	iSCAN	Utilized *trans*-cleavage activity of Cas12a	*Lachnospiraceae bacterium* (LbCas12a)	SARS-CoV-2	Non-targeted fluorophore quencher (FQ)-labeled ssDNA reporters	Fluorescence and lateral flow assay	Sensitive to SARS-Cov2 virus	System involving RT-LAMP coupled with CRISPR-Cas12, as an efficient detection module for COVID-19	1 h/2–5 USD per reaction	iSCAN can easily detect any virus strain and differentiates various strains and determine the dominance of a specific strain in a certain location or population	51
5	iSCAN-V2	Utilized *trans*-cleavage activity of Cas12b	*Alicyclobacillus acidiphilus* (AapCas12b)	SARS-CoV-2	ssDNA-labelled HEX reporter	Fluorescence	iSCAN-V2 showed a detection limit of 40 copies per μL for SARS-CoV-2	It is reliable and efficient for detecting SARS-CoV-2 RNA in patient samples	<1 h	iSCAN-V2 has high specificity with 93.75% concordance with standard RT-qPCR	52
6	CRISPR-eSPR	Utilized SpCas9 RNPs that bind to the substrate	*Streptococcus pyogenes* (SpCas9)	Duchenne muscular dystrophy	—	Surface plasmon resonance (SPR) sensor	CRISPR-SPR-Chip with a limit of detection as low as 1.3 fM	The CRISPR-SPR-Chip could distinguish single-site mutations introduced in a gene	<10 min	CRISPR-eSPR specifically detects a target gene sequence, providing a new on-chip optic approach for clinical gene analysis	53
7	CREST	Utilized collateral cleavage of the Cas13a	*Leptotrichia wadei* (LwaCas13a)	SARS-CoV-2	Fluorescein- and quencher-conjugated poly(U) RNA cleavage reporter probe	Fluorescence and lateral flow assay	It can detect 10 copies of a target RNA molecule per μL sample	The method was adopted for the detection in human swab spiked with heat-inactivated SARS-CoV-2	2 h/$0.05 and $0.50 USD per reaction	CREST could be employed for regular testing as well as for disambiguation of results obtained with established methods	54
8	SATORI	Utilized collateral cleavage activity of CRISPR-Cas13a	*Leptotrichia wadei* sp. (LwaCas13a protein)	SARS-CoV-2	ssRNA-labelled FQ reporter	Fluorescence	SATORI detected single-stranded RNA targets with maximal sensitivity of ∼10 fM in <5 min	SATORI is an accurate, rapid, and robust ssRNA detection platform	US$9.10	SATORI could be used for a more rapid and robust primary screening for infections of ssRNA viruses	55
9	CASCADE	Utilized *trans*-cleavage activity of Cas12a	*Lachnospiraceae bacterium* (Lba Cas12a)	SARS-CoV-2	ssDNA-labelled FAM reporter	Fluorescence and smart phone-based readout	High analytical sensitivity in signal detection without previous target amplification (down to 50 copies per μL) is observed in spiked samples	An amplification-free CRISPR/Cas12-based diagnostic technology for SARS-CoV-2 RNA detection	71 min	Cellphone-based analysis eliminates the requirement of specialized and bulky equipment	56
10	PPCas12 assay	Utilized *trans*-cleavage activity of Cas12a	*Lachnospiraceae bacterium* (LbCas12a)	Detection of foodborne pathogens without amplification steps	ssDNA-labelled FAM reporter	Fluorescence	The LOD of the assay was 3110 CFU	Nucleic acid amplification-free assay for detecting *S. enterica*	<1 h	PPCas12 assay will be a valuable tool for on-site monitoring of foodborne pathogens	57
11	Ultralocalized Cas13a assay	Utilized collateral cleavage activity of CRISPR-Cas13a	*Leptotrichia buccalis* (LbuCas13a)	SARS-CoV-2 RNA	ssRNA-labelled FAM reporter	Fluorescence and simple droplet microfluidic platform	This method achieved single-molecule analytical sensitivity, which is 104-fold more sensitive than those reported methods	This system may introduce a route toward simple and highly sensitive nucleic acid detection for other CRISPR-Cas systems	<1 h	Attractive alternative to achieve single-molecule sensitivity and eliminate nucleic acid amplification-related problems	58
12	FELUDA	Utilized FnCas9 RNPs that bind to the substrate	*Francisella novicida* (FnCas9)	SARS-CoV-2 and sickle cell disease (SCD)	Dead FnCas9 (dFnCas9) tagged with a fluorophore (GFP) is adept in sensing a point mutation on DNA using microscale thermophoresis	Fluorescence and lateral flow assay	FELUDA reached a limit of detection (LOD) of ∼10 copies of purified viral sequence	Simultaneous detection of SARS-CoV-2 infection as well as for detecting point mutations in the virus obtained from patient samples	1 h/7 USD for test	FnCas9 based diagnostic for detecting a wide range of pathogenic SNVs and nucleotide sequences	42
13	TwistDx	Utilized *trans*-cleavage activity of Cas12a	*Lachnospiraceae bacterium* (LbCas12a)	SARS-CoV-2	ssDNA reporters labeled with FAM	Milenia Hybri Detect 1 (TwistDx) lateral flow system and fluorescence	Sensitive to SARS-CoV-2 virus	The method was found fast, accurate for the detection of the SARS-CoV-2 sequence in patient saliva sample	90 min/$1–2 USD per reaction	CRISPR/Cas12 based detection method is characterized by a LOD value lower than the minimum levels needed to presently detect the virus in clinical samples	59
14	SHINE	Utilized collateral cleavage activity of CRISPR-Cas13a	Leptotrichia wadei sp. (LwaCas13a protein)	SARS-CoV-2	FAM quenched reporter	App-enabled in-tube fluorescence and lateral flow assay	Sensitive to SARS-CoV-2 virus	Detecting viral RNA and SHINE demonstrates perfect concordance with RT-qPCR in our samples with titers >1000 viral cp per μL	1 h	A simple method for detecting viral RNA from unextracted patient samples and multiple readouts	60
15	STOP Covid	Utilized *trans*-cleavage activity of Cas12b	*Alicyclobacillus acidoterrestris* Cas12b (AacCas12b) and *Alicyclobacillus acidiphilus* Cas12b (AapCas12b)	SARS-CoV-2	Fluorescent reporter:/5HEX/TTTTTTT/3IABkFQ/and lateral flow reporter:/56-FAM/TTTTTTT/3Bio/	Fluorescence and lateral flow assay	Sensitive to SARS-CoV-2 virus	STOP Covid, provides sensitivity comparable to RT-qPCR-based SARS-CoV-2 tests and has a limit of detection of 100 copies of viral genome input in saliva or nasopharyngeal swabs per reaction	40 to 70 min/$40 USD for test	STOP Covid is point of care platform for COVID-19 diagnostics	61
16	PECL-CRISPR	Utilizes the *trans*-cleavage activity of CRISPR/Cas13a	*Leptotrichia buccalis* (LbuCas13a)	Human breast adenocarcinoma cells and human hepatocellular liver carcinoma cells	Planar phenazine ligand of [Ru(phen)_2_dppz]^2+^	Electrochemiluminescence	MicroRNA with limit of detection 1 fM	CRISPR/Cas13a powered portable ECL chip (named PECL-CRISPR) for ultrasensitive and high-specificity analysis of miRNA	1 to 2 h	Detection of MiR-17 in cell extracts and raw cell lysates. Limit-of-detection of 1 × 10^−15^ M for miR-17	62
17	CARMEN	Utilized collateral cleavage activity of CRISPR-Cas13a	*Leptotrichia wadei* species (LwaCas13a)	Zika virus cDNA	NA	Fluorescence microscopy	It is a diagnostic platform for extraction-free viral RNA detection	CARMEN is particularly well suited for community surveillance testing, as it combines user-friendly, simple preparation methods with sufficient sensitivity and a rapid turn-around time	3 h	Detection of 169 viruses; subtyping of influenza A; detection of HIV drug resistance mutations	63
18	APC-Cas	Utilized collateral cleavage activity of CRISPR-Cas13a	*Leptotrichia buccalis* (LbuCas13a)	Bacterial pathogens detection, *e.g. Salmonella enteritidis*	Fluorescence combination of nucleic acid-based allosteric probes and CRISPR-Cas13a components	Laser scanning confocal microscope (LSCM) and electrophoretic analysis	It can selectively and sensitively quantify *Salmonella enteritidis* cells (from 1 to 10^5^ CFU) in milk	APC-Cas may have potential clinical applications for early diagnosis of pathogens	2 h 20 min/$0.86 USD for test	APC-Cas can identify low numbers of *S. enteritidis* cells in mouse serum, distinguishing mice with early- and late-stage infection from uninfected mice	64
19	E-CRISPR	Utilized *trans*-cleavage activity of Cas12a	*Acidaminococcus* species (AsCas12a) and *Lachnospiraceae* bacterium ND2006 Cas12a (LbCas12a)	Human papilloma virus (HPV) Sub type, HPV-16	Electrochemical detection micro-fabricated gold-based three-electrode sensor with gold as working and counter electrodes and Ag/AgCl as reference electrode	Electrochemical current of methylene blue	A linear detection range was achieved covering three orders of magnitude with an LOD of 0.2 nm	To detect viral nucleic acids like human papillomavirus 16 (HPV-16) and parvovirus B19 (PB-19), with a picomolar sensitivity. It was further designed for the detection of transforming growth factor β1 (TGF-β1) protein in clinical samples	1–3 h	E-CRISPR cascade was designed for protein detection and the high specificity of target recognition, more than a gene editing tool	65
20	HOLMES	Utilized *trans*-cleavage activity of Cas12a	*Lachnospiraceae* bacterium (LbCas12a)	DNA viruses *e.g. Pseudorabies* virus (PRV), and RNA viruses *e.g.* Japanese encephalitis virus (JEV)	Collateral ssDNA probe HEX-N12-BHQ1	Fluorescence	Minimum detectable concentration for Cas12a-crRNA was approximately 0.1 nM	HOLMES may have advantages in DNA detection and rapid PCR amplification was used	15 min	Shorter guide sequences and sequence-independent detection of any single nucleotide polymorphism (SNP) sites was performed through HOLMES	66
21	HOLMASv2	Utilized *trans*-cleavage activity of Cas12b	*Alicyclobacillus acidoterrestris* (AacCas12b)	Cancer	Collateral ssDNA probe (HEX-N12-BHQ1)	Fluorescence	Minimum detectable concentration for Cas12b-crRNA was approximately 1 nM and when combined with loop-mediated isothermal amplification (LAMP), the required target concentration decreased to 10^−8^ nM	Merits of high accuracy, rapid speed and much less cost by HOLMESv2; it may serve as a potential platform for efficient detection of DNA methylation degree and target DNA quantitation in one step	1 h	To detect human mRNA, viral RNA, circular RNA easily, could discriminate single nucleotide polymorphism (SNP), avoid cross-contamination, comprises one-step analysis, could quantify methylation status of target DNA	67
22	DETECTR	Utilized *trans*-cleavage activity of Cas12	*Lachnospiraceae* bacterium (LbCas12a)	Human papillomavirus (HPV)	ssDNA-FQ reporter substrate	Fluorescence	Achieves attomole sensitivity for DNA detection	A method that RNA guided DNA binding unleashes indiscriminate single-stranded DNA (ssDNA) cleavage activity by Cas12a that completely degrades ssDNA molecules	1 h	DETECTR enables rapid and specific detection of human papillomavirus in patient samples, thereby providing a simple platform for molecular diagnostics	68
23	SHERLOCK/CRISPR-Dx	Utilized collateral cleavage activity of Cas13a	*Leptotrichia wadei* (LwaCas13a)	Dengue, zika virus, HPV infection	ssRNA-labelled FAM reporter	Fluorescence	Detection sensitivity of ∼50 fM	Detection of nucleic acid using RNA-guided RNA targeting Cas13a, which was previously named as C2c2 bearing RNase activity	2–5 h/$0.61 USD for test	Capable of single-molecule DNA detection and can distinguish two strains of the same virus	69
24	SHERLOCKv2	Utilized collateral cleavage activity of Cas13a	*Leptotrichia wadei* (LwaCas13a), *Prevotella* species PsmCas13b, *Capnocytophaga canimorsus* CcaCas13b, and AsCas12a	Dengue or zika virus	FAM, TEX, Cy5, and HEX channels	Fluorescence and lateral flow assay	Sensitive to detect nucleic acid for any types of viral or non-viral infection or mutation	SHERLOCKv2 was designed considering the conditions where more than one target needs to be detected in a single reaction	90 min	It is employed to detect EGFR L858R mutation cfDNA from patient samples successfully. Later, to improve efficiency, the RPA, Cas13/Csm6, and lateral flow readout all were combined to detect the acyltransferase target	70

## CRISPRD

Recently, Ghouneimy *et al.* (2024) introduced a multiplex-point-of-care platform for detecting high-risk human papillomavirus (hrHPV). They postulated a one-pot assay by harnessing the compatibility of thermostable AapCas12b, TccCas13a, and HheCas13a nucleases with isothermal amplification. Eventually, using this assay method, they successfully detected cervical cancer-causing hrHPV16 and HPV18 along with an internal control with a detection limit of 10 copies and 100% specificity. This platform, CRISPR-Cas multiplexed diagnostic assay (CRISPRD), offers a rapid and practical solution for the multiplex detection of hrHPVs, which may facilitate large-scale hrHPV point-of-care screening. Furthermore, the CRISPRD platform can be easily reprogrammed and is customizable, and it can be recruited for the multiplex detection of nucleic acid biomarkers as well as internal control.^48^

## RPA-CRISPR-Cas12a

Qu *et al.* (2024) established a rapid, reliable, and inexpensive detection method based on CRISPR/Cas12a technology for detecting *Fusobacterium nucleatum* (Fn). Fn is a conditional pathogen that can cause various oral and gastrointestinal diseases. This assay's components include specific recombinase polymerase amplification (RPA) primer sequences and crRNA sequences specific to the nusG gene of Fn and an active Cas12a protein. As a result, a fluorescence assay and a lateral flow immunoassay were established using the RPA and CRISPR-Cas12a system (RPA-CRISPR-Cas12a). The sensitivity of this assay was found to be with a detection limit of 5 copies per μL. This method could distinguish Fn from other pathogens with excellent specificity. Furthermore, the RPA-CRISPR-Cas12a assay was highly consistent with the classical quantitative real-time PCR method when testing periodontal pocket samples. This feature makes RPA-CRISPR-Cas12a a promising technique for detecting Fn and several other pathogens.^49^

## CRISPR/Cas12a system with a digital microfluidic chip (DMF)

Lu *et al.* (2023) designed a rapid, sensitive and automated method to detect bacterial pathogens by integrating the CRISPR/Cas12a system with a digital microfluidic chip (DMF). With this assay, they achieved a detection limit of 32 CFU mL^−1^ in the case of *Staphylococcus aureus*. The sensitivity and specificity of this assay are at an exceptional level where one can perform the whole detection assay using a tiny volume (6.6 μL) of reagent with under 55 min duration. Eventually, with this excellent sensitivity and reliability, this platform can be successfully applied to several ranges of human samples from urine and milk. With automation in fluid handling of the droplets, this method can sequentially perform several biochemical assays such as cell lysis, RPA amplification and Cas12a *trans*-cleavage on a DMF chip without manual operations.^50^

## iSCAN

Ali *et al.* used LbCas12a effector protein associated with RT-LAMP to develop an efficient and rapid method named iSCAN (*in vitro* specific CRISPR-based assay for nucleic acid detection) for the detection of SARS-CoV-2. The method is composed of RT-LAMP coupled with crRNA/LbCas12a effector protein to detect target viral DNA through collateral activity. The workflow of iSCAN started with a sample collected from an infected patient, followed by isolation of total RNA. RT-LAMP was performed to get the amplified target sequence and incubated with crRNA/LbCas12a complex (where crRNA was designed for N and E gene region of the SARS-CoV-2 genome). FQ-ssDNA reporter was added to the reaction mixture and incubated at predetermined time points such as 5, 10, 20, and 30 min at 37 °C. The fluorescence signals were obtained because of collateral activity in SARS-CoV-2 infected samples after 20 min of incubation. On the other hand, no fluorescence was detected in control samples. The efficiency of iSCAN was validated using a nucleic acid sample for five patients who were found positive in RT-PCR-based assay and results showed 100% agreement. Further, the iSCAN technique was combined with lateral flow immunochromatography and the assay showed 100% concordance with previous results. Later, two variants of Cas12, *i.e.*, AacCas12b (*Alicyclobacillus acidoterrestris*) and AapCas12b (*Alicyclobacillus acidophilus*), were employed replacing LbCas12a to develop a one-pot assay, since AacCas12b and AapCas12b are thermophilic variants of Cas12b and can catalytically function in the same temperature range as RT-LAMP to minimize liquid handling. Clinical samples were tested with thermophilic variant containing iSCAN *via* both fluorescence and lateral flow-based readout. The fluorescence readout showed 18 out of 21 qPCR positive samples as positive, while 3 out of 3 negative samples as negative. On the other hand, the lateral flow-based readout results are compromised due to low fluorescence detection.^51^

## iSCAN-V2

Intending to improve on the original iSCAN, Aman *et al.* developed an updated version, *i.e.*, iSCAN-v2, in 2022. In this work, iSCAN was modified to a one-pot assay to make it advantageous for a ‘point of care’ detection system. However, the sole purpose of realizing a one-pot assay was to diminish the chance of cross-contamination during the reaction process. The assay uses reverse transcription recombinase polymerase enhancement (RT-RPA) assisted with CRISPR/Cas12b effector in one pot and at a single temperature to detect SARS-CoV-2. Primarily, to validate the assay, RT-RPA was carried out with synthetic SARS-CoV-2 RNA utilizing a different set of primers, crRNAs, and Cas effectors (Cas12a and Cas12b) in a two-pot assay. Both the effectors (Cas12a and Cas12b) were evaluated for their *cis*- and *trans*-cleavage activity through fluorescence signal to validate the specificity of designed crRNAs, wherein all the primers and crRNA showed robust outcomes.

**Fig. 5:**
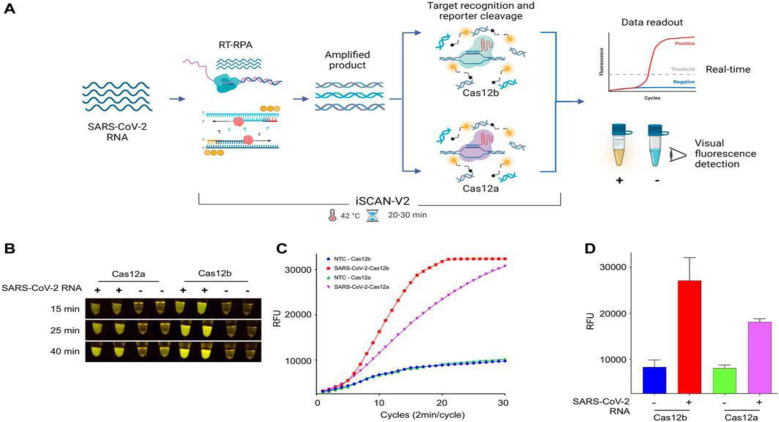
The iSCAN-V2 assay with Cas12b can efficiently detect SARS-CoV-2. (A) Schematic of CRISPR/Cas12a and Cas12b based detection of SARS-CoV-2. Total RNA was subjected to the iSCAN-V2 one-pot assay. The collateral cleavage of the ssDNA-labelled HEX reporter in the same reaction produces a fluorescent signal for visual detection. (B) iSCAN-V2-based detection of SARS-CoV-2. (C) Real-time detection of synthetic SARS-CoV-2 RNA. The iSCAN-V2 assay was performed to determine the real-time efficiency of Cas12a- and Cas12b-based target detection. Nuclease-free water was used as the no-template control (NTC). (D) Graphical representation of iSCAN-V2 end-point detection performed with CRISPR/Cas12a and Cas12b.

Overall, this method employs RT-RPA assisted with a single-guide RNA (sgRNA) and the AapCas12b effector protein to identify target viral DNA *via* collateral activity. The reaction setup started with sample collection from an infected patient, followed by total RNA isolation. Using RT-RPA, target sequences were amplified and then incubated with a crRNA/AapCas12b complex (where crRNA was designed for the N gene region of the SARS-CoV-2 genome). An ssDNA-labelled HEX reporter present in the same reaction provides a visual fluorescence signal by means of collateral cleavage and can be observed clearly after 20 min of incubation time (Fig. 5). Later, iSCAN-V2 showed a detection limit of 40 copies per μL for SARS-CoV-2. The primer set CV125F and CV434R were found to be the most efficient based on these results. As per the observations, at RNP concentrations above 110 nM, no significant improvement in the signal was detected, even after optimizing for temperature. Therefore, 110 nM of RNPs was selected as the standard concentration for all tests. A one-pot experiment was evaluated at different temperatures from 37 to 42 °C, the maximum signal being observed at 42 °C. Surprisingly, the signal intensity started decreasing at 45 °C. The Cas12b protein and the superscript IV reverse transcriptase (SSIV-RT) are stable at high temperatures, so the reduction in the signal intensity of iSCAN-V2 at 45 °C is probably due to a reduction in the performance of RPA components at high temperatures.

Further, the RNA samples extracted from COVID-19 patient swabs were used to validate the clinical translational potential of iSCAN-V2. The same samples were first evaluated for SARS-CoV-2 load using a standard RT-qPCR method approved by the Centers for Disease Control and Prevention. iSCAN-V2 reaction was used to detect 36 positive samples, 12 negative samples, and 2 no-template controls. As per the observed data, iSCAN-V2 has high specificity with 93.75% concordance with standard RT-qPCR. Collectively, the iSCAN-V2 assay was found to be time-effective (<1 h), reliable and efficient for detecting SARS-CoV-2 RNA in patient samples with a Ct value <30. Interestingly, this method may be employed for extensive diagnostics in the event of future pandemics.^52^

## CRISPR-eSPR

Zheng *et al.* (2022) developed a surface plasmon resonance (SPR) sensor that is functionalized with a layer of locally grown graphdiyne film, achieving excellent sensing performance when coupled with catalytically deactivated CRISPR-associated protein 9 (dCas9). The dCas9 protein is immobilized on the sensor surface and complexed with a specific sgRNA, enabling the amplification-free detection of target sequences within genomic DNA. The CRISPR-SPR-Chip sensor successfully analyzes recombinant plasmids with only three-base mutations with a detection limit as low as 1.3 femtomolar (fM). Real-time monitoring CRISPR-SPR-Chip is used to analyze clinical samples of patients with Duchenne muscular dystrophy with two exon deletions detected without any preamplification step, yielding significant positive results within 5 min. The ability of this novel CRISPR-empowered SPR (CRISPR-eSPR) sensing platform for rapid, precise, selective detection of a target gene sequencing provides a new on-chip optic approach for clinical gene analysis. Advantages of the CRISPR-SPR-Chip include (1) graphdiyne film that affords a better protein loading capacity, (2) augmentation of the SPR signal response was also enhanced *via* the electromagnetic field coupling effect of graphdiyne with the Au metal layer, (3) the ability of dCas9/sgRNA immobilized on the graphdiyne film on the CRISPR-SPR-Chip to distinguish and capture specific DNA sequences at low-concentrations without preamplification (less than 5 fM), and (4) demonstration pf clinical application of the CRISPR-SPR-Chip as shown by the sensitive and accurate detection of genomic DNA in patients with Duchenne muscular dystrophy.^53^

## FELUDA

**Fig. 6:**
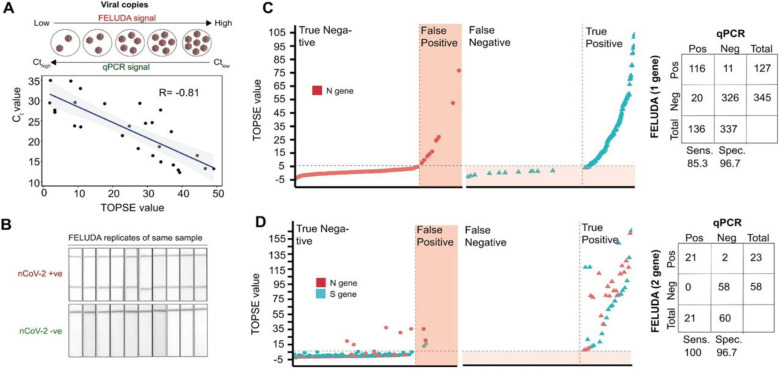
FELUDA for SARS-CoV-2 detection. (A) High concordance of FELUDA with qPCR. (B) Strong reproducibility between repeated FELUDA runs on the same positive or negative sample. (C) One gene (N gene) and (D) two genes (N and S genes) FELUDA on clinical samples on *x*-axis and distribution of TOPSE values on *y*-axis. The compiled data are in the corresponding tables on the right-hand side.

*Francisella novicida* Cas9 (FnCas9) has been used for the detection of SARS-CoV-2 infection in patient samples. Azhar *et al.* reported this method as FELUDA (FnCas9 editor linked uniform detection assay), where they utilized pre-assembled FnCas9 RNPs that bind to the substrate independent of the *trans*-cleavage activity to detect nucleotide sequence. The crRNA was designed using Web Tool JATAYU, and a FAM labeled tracrRNA was used to synthesize guide RNA followed by complexation with FnCas9 to form RNPs. The method was developed as paper strip-based technique with an absorption band on the upper end and an antiFAM antibody conjugated gold nanoparticle on the lower end. A control band appears by default when the sample is processed on the strip, while the test band appears only when the test nucleic acid sequence is detected (Fig. 6). Further, smartphone technology, *i.e.*, true outcome predicted *via* strip evaluation (TOPSE), was combined to detect the strip results, which detects the band intensity, with respect to control band. FELUDA was reported as semi-quantitative, rapid and switchable along with 100% sensitivity when employed to a lateral flow readout system. It takes 1 hour to detect any of the virus load in a clinical sample.^42^

## CREST

In 2021, Rauch *et al.* developed a scalable method for the detection of the genetic material of SARS-CoV-2. The method is named Cas13-based, rugged, equitable, scalable testing (CREST), which overcomes several existing limitations such as scalability, cost, the requirement of trained personnel and large-scale reagents. Meanwhile, CREST requires common PCR chemicals, cost-effective instruments (thermocycler) and fluorescent probes without hampering/compromising the specificity and sensitivity of the method. The overall workflow of the method starts from sample collection, extraction of RNA and reverse transcription followed by amplification using *Taq polymerase* in thermocycler conditions. Further, the transcription reaction and Cas13a activation are carried out to cleave the Poly (U) reporter, which further produces fluorescence that is visualized at 495 nm using blue LED (P51 molecular fluorescence visualizer). Later, the method was also reported as an efficient lateral flow detection system on strips. The sensitivity of CREST could be seen by its detection efficiency of 10 copies of a target RNA molecule per microlitre (μL) of sample. When the method was adopted for detection in human swab spiked with heat-inactivated SARS-CoV-2, the limit was observed at 200 copies per μL. The overall results showed that CREST has sensitivity, specificity, and accuracy of 96.9, 98, and 97.7%, respectively.^54^

## SATORI

Shinoda *et al.* (2021) developed a new CRISPR-based amplification-free digital RNA detection platform (SATORI) by combining CRISPR-Cas13-based RNA detection and microchamber array technologies. In this method, 10 crRNAs target different regions of the SARS-CoV-2 N-gene RNA. Upon target RNA binding, LwaCas13a-crRNA complexes cleave RNA targets in *cis* or *trans* form into multiple RNA fragments. Thus, using multiple crRNAs complementary to different regions of a target RNA could generate multiple LwaCas13a-crRNA-tgRNA molecules from a single target RNA molecule by increasing the potential number of positive chambers. SATORI detected single-stranded RNA targets with a maximal sensitivity of 10 fM in <5 min, with high specificity. Furthermore, the simultaneous use of multiple different guide RNAs enhanced the sensitivity, thereby enabling the detection of the SARS-CoV-2 N-gene at 5 fM levels. Therefore, SATORI will be a powerful, accurate, and rapid diagnostic method.^55^

## CASCADE

Silva *et al.* (2021) designed a cellphone-based amplification-free system with a CRISPR/CAS-dependent enzymatic (CASCADE) assay to detect SARS-CoV-2 RNA using CRISPR/Cas12a. The working principle relies on a smartphone camera imaging a catalase-generated gas bubble signal within a microfluidic channel and does not require external hardware optical attachments. There is spontaneous generation of bubble signals on catalase and ssDNA probes when a Cas12a coupled RNP complex detects and *trans*-cleavages the reverse-transcribed DNA/RNA heteroduplex of SARS-CoV-2. This event triggers a series of pervasive signals. This device offers high analytical sensitivity in signal detection without preamplification of the target (down to 50 copies per μL), which is observed in spiked samples in ≈71 min from sample input to results readout. With the aid of a smartphone vision tool, high accuracy (AUC = 1.0; CI: 0.715–1.00) is achieved when the CASCADE system is tested with nasopharyngeal swab samples of PCR-positive COVID-19 patients.^56^

## PPCas12 assay

Zhang *et al.* (2021) devised an assay to analyze pathogenic genes directly based on CRISPR-Cas12. This new test, termed proximal DNA probe-based CRISPR-Cas12 (PPCas12), facilitates the detection of foodborne pathogens without amplification steps. The substitution of the frequently used dually labeled DNA reporter with a proximal DNA probe in the PPCas12 assay led to a 4-fold sensitivity enhancement. PPCas12 offered a limit of detection of 619 colony-forming units in the detection of *Salmonella enterica* without the nucleic acid amplification process. The specificity of genes *via* PPCas12 allowed for the distinction of *S. enterica* from other foodborne pathogens. The PPCas12 assay was applied with high precision to screen *S. enterica* contamination on fresh eggs. Hence, the new PPCas12 assay will be a valuable tool for on-site monitoring of various foodborne pathogens.^57^

## Ultralocalized Cas13a assay

Tian *et al.* (2021) introduced a confinement effect-inspired Cas13a assay for single-molecule RNA diagnostics, eliminating the need for nucleic acid amplification and reverse transcription PCR (RT-PCR). This assay involves confining the RNA-triggered Cas13a catalysis system in cell-like-sized reactors to enhance target and reporter local concentrations *via* droplet microfluidics. It achieves more than 10 000-fold enhancement in sensitivity when compared to the bulk Cas13a assay and enables absolute digital single-molecule RNA quantitation. Notably, this direct RNA diagnostic technology detects a wide range of RNA molecules at the single-molecule level. Moreover, its simplicity, universality, and excellent quantification capability might make it a dominant rival to RT-qPCR. Its exceptional sensitivity, specificity, and expandable applicability are experimentally demonstrated for precisely counting microRNAs, 16S rRNAs, and SARS-CoV-2 RNA from synthetic sequences to clinical samples.^58^

## TwistDx

In 2020, the Covid pandemic provided a great opportunity for researchers to work on the detection of viral infection using the CRISPR/Cas system, and therefore much research emerged with many translation approaches. Lucia *et al.* developed an RNA-guided DNAse, *i.e.*, type V-Cas12a based, rapid, ultrasensitive, and portable method for the detection of SARS-Cov-2 infection. In brief, SARS-Cov-2 RNA fragments (10^5^ copies per μL) from saliva samples were amplified using RT-RPA to synthesize cDNA followed by incubation with LBCas12a/SARS-Cov-2sgRNA complexes (37 nM each) along with 1 μM FAM tagged ssDNA reporter. The reaction pool was kept at RT for collateral *trans*-cleavage resulting in the emission of fluorescence. The method was developed in two ways. One is based on fluorescence detection using a plate reader at excitation and emission wavelengths of 485 nm and 535 nm, respectively, using a SpectraMax M2 fluorescence plate reader (Molecular Devices) for 10 min intervals. The second method is based on paper-based measurement using a Milenia 91 HybriDetect 1 (TwistDx) lateral flow system. The method was found to be fast and accurate for the detection of the SARS-CoV-2 sequence in patient saliva samples.^59^

## SHINE

Despite being a sensitive and potential tool for nucleic acid detection, the existing method required modification for scalability and clinical translation. One such example is specific high-sensitivity enzymatic reporter unlocking (SHERLOCK), which utilizes multistep processing started with isolated nucleic acid followed by isothermal RPA, T7 transcription, and incubation with Cas13 effector to get a fluorescent signal *via trans*-cleavage activity in the reporter. However, the area of CRISPR/Cas-based biosensors is growing swiftly, and the current SHERLOCK methods are compatible with HUDSON, which deactivates nucleases and lyses viral particles through the application of heat and chemical reduction and therefore eliminates the nucleic acid extraction step to make the process less time-consuming. The necessity to transfer amplified products between tubes, which are prone to error and misinterpretation of results, limits the scalability and widespread deployment of these methods. To overcome such limitations, Arizti-Sanz *et al.* developed a specific and sensitive diagnostic tool, *i.e.*, streamlined highlighting of infections to navigate epidemics (SHINE), by using Cas13 effector to detect SARS-CoV-2 RNA from unextracted samples. Briefly, firstly, to reduce time and user error, a SHERLOCK-based SARS-CoV-2 assay was established to combine the amplification step and the *trans*-cleavage-based detection step. Secondly, when the HUDSON treated SARS-CoV-2 RNA sample was detected using SHINE, better results were observed. As per the colorimetric readout data, SHINE was able to detect ∼50 copies per μL of SARS-CoV-2 RNA from the universal viral transport medium (UTM), while the limit was 100 copies per μL in saliva samples. Similarly, as per the fluorescent readout data, which take ∼1 h to collect, SHINE was able to detect HUDSON-treated SARS-CoV-2 RNA with a limit of 10 cp per μL, 5 cp per μL and 5 cp per μL in UTM, viral transport media (VTM) and saliva, respectively. Additionally, a smartphone app that leverages a built-in camera to photograph illuminated reaction tubes was developed to minimize user bias in reading the in-tube output. Collectively, SHINE was found to be sensitive, specific, and precise in terms of nucleic acid detection.^62^

## STOP-Covid

Julia Joung *et al.* developed simple test chemistry called STOP (SHERLOCK testing in one pot) for detecting SARS-CoV-2 in one hour that is suitable for point-of-care use. STOP-Covid, a simpler SARS-CoV-2 test, has a detection limit of 100 copies of viral genome input in saliva or nasopharyngeal swabs per response, which is comparable to RT-qPCR-based SARS-CoV-2 assays. The test takes 70 minutes to complete using lateral flow readout and 40 minutes to complete using fluorescence readout. Furthermore, COVID-19 patient nasopharyngeal swabs were tested using STOP-Covid and it was found that it properly diagnosed 12 positive and 5 negative patients out of three replicates. STOP-Covid is a suitable platform for creating point-of-care COVID-19 diagnostics, and it has the potential to play a significant role in implementing effective test-trace-isolate strategies to stop the COVID-19 outbreak.^61^

## PECL-CRISPR

In 2020, Zhou *et al.* developed a miRNA detection technique named CRISPR/Cas13a powered portable electrochemiluminescence chip (PECL-CRISPR) by utilizing RNA recognition and signal amplification abilities of Cas13a effector protein. The technique offers more sensitivity by means of a decrease in background noise. PECL-CRISPR was further utilized to develop point-of-care detection by making it a paper chip-based biosensor. In a nutshell, the target miRNA induces Cas13a to cleave a well-designed pre-primer, triggering exponential amplification and ECL detection. The method was able to detect miRNA-17 with a minimum range of 1 × 10^−15^ M. Interestingly, PECL-CRISPR was able to differentiate the concerned miRNA from its homologous family members, demonstrating a high level of resolution against single nucleotide changes. Additionally, to make this diagnostic system more feasible and straightforward, a light switch [Ru(phen)_2_dppz]^2+^ method was introduced, wherein an oxidation reaction occurs between DNA and [Ru(phen)_2_dppz]^2+^ complex leading to the generation of the signal at a BPE anode and is directly proportional to the miRNA concentration. The introduction of [Ru(phen)_2_dppz]^2+^ makes the detection process quantitative as well as smooth by avoiding the washing step. Furthermore, the application of the method was evaluated by detecting miRNA-17 in cancer cells (MDA-MB-231, MCF-7, HepG2). For validation of the PECL-CRISPR method, RT-PCR-based detection was taken as standard. As per the results, the expression of miRNA-17 was found to be significantly higher in all three cancer cell lines with respect to control cells (*i.e.*, LO2 cells). Collectively, the method was found to be precise, cost-effective, less tedious, and specific for the detection of miRNA in different cell lysates and could be potentially adopted as a molecular diagnostic tool.^62^

## CARMEN

Combinatorial arrayed reactions for multiplexed evaluation of nucleic acids (CARMEN), a platform for scalable, multiplexed pathogen detection, was developed by Ackerman *et al.* In the CARMEN technology, nanoliter droplets containing CRISPR-based nucleic acid detection reagents self-organize in a microwell array, coupling with droplets of amplified samples and allowing each sample to be analyzed in triplicate against each CRISPR RNA (crRNA). The combination of CARMEN and Cas13 detection (CARMEN-Cas13) enables the accurate evaluation of over 4500 crRNA-target pairings on a single array. A multiplexed test based on CARMEN-Cas13 was developed that differentiates all human-associated viruses with at least 10 known genome sequences while also fast integrating an additional crRNA to detect the COVID-19 pandemic's causative agent. CARMEN-Cas13 also allowed for multiplexed identification of hundreds of HIV drug-resistance mutations as well as thorough influenza A strain subtyping.^63^

## APC-Cas

Despite nucleic acid detection, CRISPR has opened up the scope for the detection of pathogens in blood samples with high specificity. An allosteric probe catalysis and CRISPR-Cas13a amplification process, named as APC-Cas, was introduced by Shen *et al.* The technique was able to detect pathogens in blood without any extraction/isolation process with a sensitivity of 1 to 10^5^ CFU. The method comprises three-step amplification reactions followed by the production of a fluorescent signal, which gives a quantitative measurement of the pathogen present in the sample. The sensitivity of the method was confirmed by using *Salmonella enteritidis* cells, and as per the observation, the fluorescence intensity increases proportionally. Moreover, to confirm the specificity of the method, pathogens, *i.e.*, *Escherichia coli*, *Staphylococcus aureus*, and *Listeria monocytogenes*, were selected and analyzed using APC-Cas. As per the results of the experiment, even in the presence of 1 × 10^3^ CFU of *Escherichia coli*, *Staphylococcus aureus* and *Listeria monocytogenes*, no fluorescence signal was observed while the fluorescence signal was 10-fold higher in the case of *Salmonella enteritidis* with the same number of cells. Further, APC-Cas was validated by detecting *Salmonella enteritidis* in milk samples and compared with the standard RT-PCR method, wherein it showed positive results for all the milk samples (*p* < 0.0001) with respect to RT-PCR sample (*p* < 0.01). Additionally, APC-Cas can also detect modest amounts of *Salmonella enteritidis* cells in mouse serum, allowing researchers to discriminate between infected and uninfected animals. Collectively, the method was found to be sensitive, selective, or specific for the detection of pathogens in biological samples and could provide early-stage diagnosis of bacterial infection.

Although APC-Cas is carried out in three cycles with three separate enzymes and must be designed and manufactured for different targets, it does not require bacterial isolation, nucleic acid extraction, or washing processes that other pathogenic bacteria detection techniques require. This method also has the advantages of a rapid test time (140 minutes), high sensitivity, and minimal sample preparation (2.5 μL). The APC-Cas reagents are very inexpensive, costing as little as $0.86 for each test, and the system may be used to detect a variety of bacteria and macromolecules.^64^

## E-CRISPR

In 2019, Dai *et al.* developed an electrochemical biosensor utilizing type V CRISPR effector protein (Cas12a, Cpf1) named as E-CRISPR for the early-stage diagnosis of diseases through nucleic acid detection. Interestingly, this was the first study that explored electrochemical biosensors, which possess advantages such as portability, specificity, swiftness, and cost-effectiveness over the existing optical transduction-based biosensors. In the principle of the technology, the *trans*-cleavage activity of the Cas12a effector protein was utilized to cleave ssDNA reporter conjugated with an electrochemical tag on three electrode-based disposal sensors. The developed method showed a picomolar sensitivity for the detection of nucleic acids such as human papillomavirus 16 (HPV-16) and parvovirus B19 (PB-19) and was also able to differentiate them. Interestingly, the Cas12a effector required the TTTN PAM sequence to find the L1-encoding gene of HPV-16. To improve the utility of the test performance, a collateral chip-based assay was developed. Briefly, the crRNA and AsCas12a duplex was incubated with HPV-16 to form crRNA/AsCas12a/HPV-16 triplex followed by incubation with a ssDNA-reporter coated electrode. Upon reaction, the methylene B signal was measured using square wave voltammetry (SWV), which was only reduced in the presence of the cognate target with the appropriate AsCas12a-crRNA. The test takes only 1–3 h time for the detection of nucleic acid in the test sample. Later, an E-CRISPR cascade that utilizes aptamers to detect transforming growth factor beta 1 (TGF-β1) protein in clinical samples was also developed. Conclusively, E-CRISPR was found to be a portable, accurate, less time-consuming, and cost-effective point-of-care diagnostic system with predetermined sensitivity.^65^

## HOLMES

Zetsche *et al.* explored Cpf1 (Cas12a), a type V-A CRISPR/Cas system, for its single RNA-guided endonuclease lacking tracrRNA, and utilizes a T-rich protospacer adjacent motif. Moreover, Cpf1 cleaves DNA *via* a staggered DNA stranded break.^72^ Utilizing this feature of Cpf1, Li *et al.* chose a Cpf1 target ssDNA probe, namely HEX-N12-BHQ1, having ssDNA with quenched fluorescence. The hypothesis was that if the Cpf1 complex (Cas12a/crRNA) *trans*-cleaved the non-targeted ssDNA, it will produce HEX fluorescence and confirm the presence of target DNA/RNA. This technique was named as a one hour low-cost multipurpose highly efficient system (HOLMES). In brief, the targeted DNA was PCR-amplified followed by incubation with binary complex (Cas12a/crRNA) along with an ssDNA probe. The reaction was incubated for 15 min at 37 °C temperature. The presence of the targeted sequence led to the *trans*-cleavage resulting in strong fluorescence from the ssDNA probe. HOLMES also showed attomole sensitivity with definite sensitivity.^66^

## HOLMESv2

In 2019 Li *et al.* presented an advanced version 2.0 of HOLMES (*i.e.*, HOLMESv2) comprising Cas12b-based detection of nucleic acid. The basic idea of HOLMESv2 is in line with the previous version of HOLMES with a modification in terms of employing a thermophile CRISPR effector protein, AacCas12b, from *Alicyclobacillus acidoterrestris*. The *trans*-cleavage activity of Cas12 was utilized to produce a nucleic acid detection assay with advantages: detecting human mRNA, viral RNA, circular RNA easily and discriminating single nucleotide polymorphism (SNP), avoiding cross-contamination, using one-step analysis and quantifying the methylation status of target DNA. The novelty of the work lies within its application in nucleic acid detection along with epigenome modification (degree of DNA methylation) analysis. Briefly, Cas12b/crRNA binary complex along with DNA probe incubated with target sample (ssDNA, dsDNA, non-target DNA) results in a *trans*-cleaved product that produces target-specific fluorescence. ssDNA, *i.e.*, DNMT-1, and GAPDH mRNA were tested to evaluate the functionality of HOLMESv2 for DNA and RNA, respectively. Cancer-specific cdr1s circular RNA was also detected successfully in HEK293 cells using HOLMESv2.

Further, the collagen α2(I) (COL1A2) gene, which is involved in various cancers, was used for determination in a degree of methylation assay. Firstly, two fragments of COL1A2 gene, *i.e.*, COL1A2 (BSP)-C and COL1A2 (BSP)-T, which covered the M3 site, were cloned and PCR amplified, which mimicked bisulfite-treated samples with 0% and 100% methylated status, respectively. These two fragments were mixed in distinct proportions and incubated with HOLMESv2 (sgRNA-COL1A2m3-C12 and Cas12b complexes) to get a standard curve. Thereafter, the genomic DNA from 293T, SW480, NCI-N87, and MCF cells was isolated, treated with disulfide, followed by PCR amplification of the M3 site and HOLMESv2 assay. The quantification of methylation status was done utilizing a standard curve. Like HOLMES version 1, here also the presence of target DNA/RNA sequence led to fluorescence for detection specifically within a one-hour period. Various existing methods were used to calibrate or confirm the results and the data suggested similar outcomes. Conclusively, HOLMESv2 could be made even more sensitive by combining it with either recombinase polymerase amplification (RPA) or isothermal loop-mediated isothermal amplification (LAMP). After that, it could be adopted as a standard method for detecting nucleic acids.^67^

## DETECTR

In 2018, Jennifer Doudna utilized the collateral activity of Cas12a effector protein to develop a novel method named DNA endonuclease targeted CRISPR *trans* reporter (DETECTR) for diagnostic application. In this method, the author explored the nonspecific activity of Cas12a to degrade adjacent DNA after the recognition of target DNA. The effector protein, *i.e.*, Cas12a, was obtained from the *Lachnospiraceae* bacterium (LbCas12a). Briefly, a crRNA was designed according to the predetermined sequence of target and complexed with LbCas12a to form a duplex. Further, a ssDNA reporter was used as a fluorescent probe. All three components, *i.e.*, ssDNA probe, crRNA, LbCas12a, were incubated with a biological sample containing target nucleic acids. Upon finding the complementary sequence of nucleic acids, the LbCas12a performs the collateral activity to degrade the DNA probe reported to emit fluorescence as an indication of the presence of the target in the biological sample. The fluorescence could be detected using a fluorometer. DETECTR was found to be efficient in the detection of HPV infection, wherein the PCR-detected samples were reconfirmed using DETECTR and the DETECTR successfully identified 25 out of 25 samples of HPV18 strain while 23 out of 25 samples of HPV16 strain. Additionally, the DETECTR method takes only 1 h for detection and is also capable of differentiating strains 16 and 18 of HPV easily.^68^ In 2020, Zhao *et al.* reported a unique, sensitive, and rapid method for the detection of exosomes utilizing Cas12a mediated *cis*- and *trans*-cleavage feature. The research by Zhao *et al.* led to the development of a DNA endonuclease-targeted CRISPR *trans* reporter (DETECTR) system that can achieve attomole-level single-base detection of the nucleic acid molecules. Briefly, CD63 aptamer was designed and complexed with a blocker with complementary DNA strand. The complex of CD63 aptamer and blocker was used to detect CD63 bearing exosome since the interaction of aptamer with exosome bearing CD63 leads to a conformation change in an aptamer, and the blocker gets released from the aptamer. Furthermore, CRISPR/Cas12a recognized the released blocker and carried out a non-specific *trans*-cleavage activity towards the TaqMan probe (coupled with Cy3 fluorochrome and blank hole quencher), resulting in a fluorescence signal that would be directly proportional to the recorded exosomes. The technique was used to detect exosomes for culture cells as well as the plasma exosomes in the range of “10^3^ to 10^7^” particles per microliter by means of the *trans*-cleavage property of Cas12a.^73^

Additionally, a CRISPR/Cas system, *i.e.*, Cas12a/b, was also used in the field of epigenome modification, wherein Cas12a/b effector protein is utilized to analyze the methylation status of DNA/cfDNA/nucleic acid. Since the methylation status of cfDNA or nucleic acid is strongly correlated with disease state, efficient diagnosis methods were adopted/developed using a combination of epigenome modification and the CRISPR/Cas12a/b system. Doudna's lab in 2016 published experimental evidence of the CRISPR/Cas system for nucleic acid detection application. Herein, the group explored CRISPR/Cas class 2 Type VI C2c2 effector protein for its RNA-guided RNase activity. The research group stated that the non-specific cleavage activity performed by C2c2 in the *trans* substrate, upon recognition of target RNA, can be used to identify the target RNA from a pool of transcripts. When they incubated LbuC2c2 complexed with crRNAs targeting bacteriophage λ with total HeLa cell RNA, they found a strong deviation in the substantial crRNA specific fluorescence within 30 min after the reaction. In relation to the positive control, the negative controls showed negligible fluorescence and consolidated the specificity of the experiment of applicability of C2c2 for RNA detection as diagnostic.^74^

## SHERLOCK/CRISPR-Dx

Later, Gootenberg *et al.* demonstrated a rapid and sensitive method for nucleic acid detection for disease monitoring. The study aimed to detect nucleic acid using RNA-guided RNA targeting Cas13a, which was previously named as C2c2 bearing RNase activity.^69^ They have developed a CRISPR-Dx (CRISPR-based diagnostic) tool using Cas13a effector protein and named it as specific high sensitivity enzymatic reporter unlocking (SHERLOCK), which is sensitive for detection of nucleic acids at attomole concentration. The sensitivity and efficiency of SHERLOCK were demonstrated by detecting dengue and zika virus strains and cell-free DNA mutations in cancer. In brief, the DNA or RNA from the strain was isolated and processed with recombinase polymerase amplification (RPA) followed by T7 transcription. The obtained amplified product was incubated with Cas13a complex (containing Cas13a, crRNA and ssRNA reporter), where Cas13a causes collateral cleavage of the target nucleic acid to produce a fluorescence signal. Cas13a from *Leptotrichia wadei* (LwCas13a) was used that displays greater RNA-guided RNase activity relative to *Leptotrichia shahii* Cas13a (LshCas13a). The method was found capable of single-molecule DNA detection and can distinguish two strains of the same virus with a detection sensitivity of ∼50 fM. Collectively, the Cas13a mediated SHERLOCK diagnostic system was found to be highly specific, convenient, selective, and sensitive for nucleic acid detection.^69^

## SHERLOCKv2

Following the success of SHERLOCK, Gootenberg *et al.* proposed Version 2.0 of SHERLOCK (SHERLOCKv2). The basic technique was the same as that of version 1.0 with some modifications: detection range down to 2 attomol, 4-channel single-reaction multiplexing, use of auxiliary CRISPR associated enzyme (Csm6) in combination with Cas13 to improve the sensitivity up to 3.5-fold, and lateral flow readout. SHERLOCKv2 was designed considering the conditions where more than one target needs to be detected in a single reaction. Therefore, unique cleavage preference properties of different Cas effectors were utilized to create a multiplex platform system.

To determine the uniqueness in cleavage preferences, 14 members from Cas13b family and 3 members from Cas13a family were screened. The results showed that the LwaCas13a, CcaCas13b, LbaCas13a and PsmCas13b effector proteins could independently cleave di-nucleotide reporters AU, UC, AC, and GA, respectively. Now, utilizing such a unique preference for cleavage, one could detect multiple targets in the same reaction using different fluorescent ssDNA reporters in separate fluorescence detector channels. Based on this hypothesis, four different targets with different fluorescent reporters for LwaCas13a, PsmCas13b, CcaCas13b, and AsCas12a were detected in FAM, TEX, Cy5, and HEX channels, respectively. This multiplexing detection system makes SHERLOCKv2 unique in diagnostic applications. To make the technique more convenient, a lateral flow readout method was employed wherein the destruction of FAM tagged biotin reporter leads to detection on the strips, abundant reporter accumulates anti-FAM antibody-gold nanoparticle conjugates at the first line on the strip, preventing binding of the antibody-gold conjugates to protein A on the second line; cleavage of reporter would reduce accumulation at the first line and result in signal on the second line. The technology was employed to detect EGFR L858R mutation cfDNA from patient samples successfully. Later, to improve efficiency, the RPA, Cas13/Csm6, and lateral flow readout were combined to detect the acyltransferase target. The results confirmed the usefulness of Csm6 in rapid lateral flow readout with less background. Overall, SHERLOCKv2 could be employed effectively for nucleic acid detection for any type of viral or non-viral infection or mutation.^70^

## Harnessing nanotechnology to improve feasibility of CRISPR diagnostics

Nowadays, researchers mainly focus on CRISPR-based diagnostics, which originated from gene-editing technology. These are diagnostic methods developed in less than five years. These techniques have unique features like low reaction temperature, high detection specificity, and sound signal propagation that helps in the development of next-generation diagnostic tools. SHERLOCK, DETECTR, and HOLMES are CRISPR detection tools that need multiple steps in their operations like nucleic acid extraction and multiplication. In these approaches, CRISPR detection is performed independently, which results in a chance of cross-contamination, leading to difficulties in the detection process. In this present situation, most of the CRISPR diagnostic methods are used for specific detection only. They could be beneficial in precisely detecting the targeted copies. Quantitative detection systems are rare, but they have a more comprehensive range of uses in medical diagnostics as the diagnostic tool's cost and sensitivity, ease of use, and detection efficacy are essential. In short, advancements in CRISPR diagnostic procedures are crucial for their eventual development. Micro-/nanotechnology has been implemented by introducing nanochips and nanodevices into CRISPR detection methods to improve CRISPR-based nucleic acid detection. Microfluidic technology and lateral flow strips have opened up new avenues for CRISPR-based diagnostic assay design, including equipment-free detection, creating more feasible detection assays, and improving the response and quantification capabilities.

In the past few years, CRISPR detection has mainly depended on fluorescence techniques; it is more sensitive, but requires excitation and detection modules, making it challenging to utilize in point-of-care settings. Various nanoprobes like gold nanoparticles (AuNPs), platinum nanoparticles (PtNPs), copper nanoclusters, nanoflowers, and metalorganic frameworks (MOFs) are employed in CRISPR detection techniques. AuNPs and PtNPs nanoprobes generate promising results among these materials; these results can be seen with the naked eye. AuNPs have an advantage over fluorescent dye molecules because they can be seen with the naked eye at nanomolar concentrations. Their extinction coefficient is higher than those of fluorescent dye molecules. Furthermore, a change of color from red to blue-violet is seen when AuNPs shift from a scattered to an aggregated form. The redshift of AuNP plasmon resonance absorption is responsible for this alteration. The dispersion and aggregation of molecules depend on the DNA/RNA that is used for identification.^75^

In 2021 Zhang *et al.* developed a CRISPR-Cas12a colorimetric assay combined with a reverse transcription recombinase polymerase amplification (RT-RPA) to diagnose SARS-CoV-2. In this methodology, researchers used DNA-modified gold nanoparticles for the colorimetric detection of SARS-CoV-2 genome ORF1ab and N sections. By using RT-RPA, the desired dsDNA was produced and targeted with Cas12a. Due to the *trans*-cleavage activity of Cas12a, the capped DNA substrates get hydrolyzed moderately from AuNPs, resulting in a change in the surface plasmon resonance (SPR). This can be seen with the naked eye or by using UV-visible absorption spectroscopy.^76^ Further, CRISPR/Cas12-assisted RT-LAMP was designed by utilizing high-throughput gold nanoparticles for sensitive and on-site detection of SARS-CoV-2. This methodology combines two cross-linked DNA-modified AuNP probes with a linker-ssDNA, resulting in a color shift. To amplify the unique sequence RNA of SARS-CoV-2 they utilized an RT-LAMP primer targeting the N gene and the designed Cas12a/gRNA binds to the amplicon sequence and breaks down the linker-ssDNA due to the Cas12a enzyme's *trans*-cleavage activity. As a result, the AuNP probes were unable to cross-link and remained red. But in the absence of virus RNA, the designed Cas12a/gRNA failed to bind the amplicon sequence, the linker-ssDNA hybridized and cross-linked with AuNP-DNA resulting in a change of color from red to purple that could be seen with the naked eye or measured using the UV-visible spectrum. AuNP-based colorimetric test combined with Cas12a and RT-LAMP is the new technique for on-site COVID-19 diagnosis.^77^

Yuan *et al.* have also introduced a CRISPR/Cas12a/13a system for universal and naked-eye gene detection. The *trans*-cleavage activity of Cas12a/crRNA and Cas13a/crRNA on an AuNPs-DNA probes pair will help generate signals for nucleic acid detection. Briefly, when the target sequence is in the sample, the Cas12a or Cas13a effector degrades the ssDNA or ssRNA utilizing *trans*-cleavage activity. Subsequently, the hybridization linker of the AuNPs-DNA probe pair diminishes leading to no colorimetric observations. On the other hand, when there is no target sequence in the sample, the linker ssDNA and linker ssRNA are unaffected, leading to the aggregation of linker ssDNA or ssRNA with AuNPs-DNA probes. This subsequently results in colorimetric observation, which indicates a negative test signal. Further, this method can potentially be used to detect bacteria using 16S rDNA or 16S rRNA in a rapid and low-cost manner.^78^

Wang *et al.* introduced the CRISPR/Cas system into a lateral flow assay (CASLFA) to detect nucleic acids. To work with the DNA unwinding-based hybridization experiment, an AuNP-DNA probe was formed based on polyadenine (A)-Au affinity. A labeling area, a control line hybridization region, and a signal probe hybridization region are all present in the DNA sequence utilized to make the AuNP-DNA probe. Genomic material was amplified using PCR or isothermal amplification, such as RPA with the help of biotinylated primers. The biotinylated amplicons were then dripped onto the sample pad after a brief incubation with the designed Cas9/sgRNA for binding. At first, the complexes move laterally under capillary force towards the conjugate pad, where the AuNP-DNA probes hybridized with the Cas9/sgRNA-biotinylated amplicons, which resulted in the formation of an AuNP-DNA probe, biotinylated amplicon and sgRNA/Cas9 RNPs. The resulting complexes were captured by streptavidin that had been pre-coated on the test line. Excess AuNP-DNA probes flow through the lateral-flow device and are captured by hybridizing with pre-coated DNA probes at the control line. The build-up of AuNPs will result in the formation of a colorful band. The test strip detection process can be completed in 3 minutes. CASLFA works effectively for the EGFP gene employed as a detection target validating the efficacy of the DNA unwinding-based hybridization experiment.^79^

Li *et al.* designed a novel plasmonic CRISPR/Cas12a assay for the visual, colorimetric detection of grapevine viral infections. The nucleic acids are extracted by using standard virus-specific primers and PCR amplification. Cas12a protein and CRISPR RNA (crRNA) activate their specific ssDNase activity to achieve sequence-specific identification. Two DNA-functionalized AuNPs, AuNP-A and AuNP-B, are connected by a single-stranded substrate (S) linker. The cross-linked AuNPs can cause the pairing of their separate localized plasmon fields and proceed to red-to-blue color change. In the presence of the viral genetic marker and subsequent PCR amplicon, a single-stranded substrate (S) will be demolished by Cas12a, leaving the AuNP solution red. As a result, the presence (red) or absence (blue) of the viral infection could be evaluated simply by looking at the color of the assay solution with the naked eye.^80^

Hu *et al.* proposed AuNP-based bio probes to strengthen CRISPR-based diagnostics. Utilizing the thiol-free freezing method, in a single step an AuNP bio probe will be formulated, which is free from salt ageing. In recent studies, DNA-AuNP probes were prepared by a freezing-based labeling approach. It is quick but still requires thiol modification. In this technique, researchers used the thiol-free freezing-based labeling approach to evaluate a poly(A)-tagged DNA sequence, and results showed that 10 A bases are required at the sequence ends. In some cases, ineffectual or malfunctioned marking was observed because some DNA sequences create secondary structures leading the shield to expose the A bases. However, DNA sequences get adequate labeling by increasing the poly(A)-base number. Based on these findings, three types of AuNP-based bio probes, DNA-AuNP, RNA-AuNP, and DNA-enzyme-AuNP, were prepared using a freezing-based labeling approach. The AuNP probe-based magnetic pull-down method is employed to achieve colorimetric detection. Streptavidin-coated magnetic beads pull down the DNA-AuNP probe when the Cas12a system is strongly associated with *trans*-cleavage substrate biotinylated DNA. The biotinylated DNA was *trans*-cleaved by the Cas12a system that leads to escape of the DNA-AuNP probe to move in the presence of a magnetic field and red AuNPs show in the supernatant solution.^81^

Nanocatalysts like platinum nanoparticles (PtNPs) can generate oxygen from hydrogen peroxide (H_2_O_2_). By monitoring this catalytic gas production process using PtNPs as a probe, direct visual qualitative detection can be realized for this process. In 2020, Shokr *et al.* performed a virus diagnosis using a CRISPR-Cas9 system with PtNP nanoprobe with a sandwich-like recognition principle. The biotinylated target DNA/CRISPR-Cas9/anti-Cas9 antibody-coated PtNP ternary complex is generated and transferred into the chip by streptavidin-modified magnetic beads. Here, the fusion of metal nanocatalyst with target-specific recognition antibodies produces the desired signal. Mobile phones can picture and analyze oxygen production induced by PtNP, which has aided in developing a mobile health platform.^82^ Shao *et al.* utilized manufactured magnetic beads with single-stranded DNA (ssDNA)-PtNP probes (BDNPs) as CRISPR-Cas12a *trans*-cleavage substrates in another investigation. Cas12a *trans*-cleavage activity was activated by the target DNA, resulting in the degradation of ssDNA and the release of PtNPs. The movement of red ink in the microchannel was driven by the oxygen created by the released PtNPs, which could be seen with the naked eye. All of these operations were carried out on a V-chip with embedded magnets.^83^

CRISPR diagnostic approaches are more sensitive when using nanoprobes based on the metal-enhanced fluorescence (MEF) mechanism. When the fluorophore-metal contact is at an ideal distance, the fluorophore's quantum yield and light stability are greatly improved. Choi *et al.* used fluorescein isothiocyanate (FITC)-modified dsDNA and ssDNA to connect 20 and 60 nm AuNPs to construct an intelligent MEF probe system. After identifying the target DNA, the CRISPR-Cas12a performs *trans*-cleavage activity to degrade the associated ssDNA, releasing 20-AuNP-dsDNA-FITC, which luminesces due to the MEF effect. This method yielded quantitative, selective, and amplification-free cfDNA readings in buffer solution and human serum samples with sub-femtomolar sensitivity.^84^ Lee *et al.* utilized methylene blue (MB)-conjugated Au nanoparticles (MB-AuNPs) with a CRISPR system and electrochemical assay to detect the dengue virus (DENV). They have used CRISPR/Cpf1 from prevotella and francisella 1, having Cpf1 enzymatic protein, and CRISPR RNA (crRNA) recognizes a target sequence and cleaves the nonspecific single-stranded DNA (ssDNA), randomly. This method was highly sensitive and detects DENV-4 at as low as 100 fM, without any RNA amplification.^85^

In 2021, Chen *et al.* developed a CRISPR/Cas9 triggered ESDR system with a 3D GR/AuPtPd nanoflower electrochemical sensing platform for effective detection of circulating tumor DNA (ctDNA) in low levels. By combining CRISPR/Cas9 cleavage-triggered entropy-driven strand displacement reaction (ESRD) resulted in amplification efficiency and high specificity performance in efficient detection of ctDNA. This method was successfully used for the detection of EGFR in human serum.^86^ Bogers *et al.* introduced fluorescence-based diagnostic tools for ease of use, sensitivity and rapid results. This study combined CRISPR-Cas12a and terminal deoxynucleotidyl transferase (TdT) with copper nanoparticles (CuNPs) and they named this detection assay Cas12a activated nuclease poly-T reporter illuminating particles (CANTRIP). After specific target recognition by Cas12a, ssDNA reporter oligos were cut into small fragments. These act as a scaffolds for the formation of CuNPs. CuNPs produce bright fluorescent signal upon UV excitation.^87^

Nucleic acid detection methods are pivotal for pathogen detection and genotyping. Solid-state nanopore sensors are promising platforms for nucleic acid detection for their higher sensitivity and label-free electron sensing of single molecules. Nouri *et al.* developed and optimized solid-state CRISPR/Cas12a assisted nanopores (SCAN) for detection of HIV-1 DNAs. SCAN would provide a compact, rapid, and low-cost method for nucleic acid detection at the point of care.^88^ Yang *et al.*, in 2018, used solid-state nanopores to detect the DNA on CRISPR-dcas9. The variant employed here does not cut DNA but instead remains tightly bound at a used defined binding site, thus providing significant loci for biosensing. This method can be employed in other biosensing approaches like quick disease strain DNA identification and antibiotic-resistance DNA detection.^89^ Different nanocarrier-based approaches used in CRISPR-based diagnostic are shown in Table 2.

**Table 2:** Different types of nanoparticle carriers used in CRISPR diagnostics

No.	Platform for detection	Type of CRISPR system associated with nanoparticle carriers	Target analyte	Sensitivity	Time	Advantages	Reference
1	Colorimetry assays	RT-RPA coupled CRISPR-Cas12a with DNA-modified gold nanoparticles (AuNPs)	SARS-CoV-2 RNA	1 copy of viral genome sequence per test	60 min	A change in the surface plasmon resonance (SPR), it can be facially monitored by UV-vis absorbance spectroscopy and naked eye observation	76
		Cas12a assisted RT LAMP/AuNP (CLAP) assay	SARS-CoV-2 RNA	4 copies of SARS-CoV-2 RNA	40 min	Extended to high-throughput test by using a common microplate reader	77
		CRISPR-Cas12a/Cas13a assisted with AuNPs-DNA probes	Gene detection of transgenic rice, African swine fever virus	Not reported	60 min	Use of low-speed centrifugation can assist in developing a signal-on colorimetric gene detection platform with improved detection sensitivity	78
		Cas12a with plasmon coupling of DNA functionalized gold nanoparticles	Grapevine red-blotch viral infection	10 attomole to 1 picomole	—	A novel plasmonic CRIPSR Cas12a assay that generates a visual, colorimetric readout	80
2	Lateral flow assays	FnCas9 editor linked uniform detection assay (FELUDA) with gold nanoparticles (AuNPs)	SARS-CoV-2 RNA	FELUDA shows 100% sensitivity and 97% specificity across all ranges of viral loads	60 min	This technique enables a smartphone application: true outcome predicted *via* strip evaluation (TOPSE)	42
		CRISPR-Cas13 system with gold nanoparticles (AuNPs)	Dengue or zika virus ssRNA	Quantitative measurement of input as low as 2 attomoles	<90 min	Single molecule quantitation and enhanced signal with SHERLOCK and Csm6	70
		CRISPR/Cas9 system with AuNPs-DNA probes	*Listeria monocytogenes*, genetically modified organisms (GMOs), and African swine fever virus (ASFV)	100 copies of genome samples	40 min	CASLFA method can meet the needs for rapid genetic detection in most cases	79
3	Electrochemical assays	CRISPR/Cpf1 system with methylene blue (MB)-conjugated Au nanoparticles (MB-AuNPs)	DENV-4 RNA	100 femtomolar	—	The MB-AuNPs were easily functionalized on the Au microelectrode *via* SH-ssDNA-biotin for amplifying the electrochemical signals without an additional enhancing agent	85
		A novel 3D GR/AuPtPd nanoflower sensing platform based on CRISPR/Cas9 cleavage-triggered entropy-driven strand displacement reaction (ESDR)	For the effective detection of circulating tumor DNA-EGFR	0.13 picomolar	—	Powerful method has great potential for application in clinical molecular diagnosis and provides a novel approach for liquid biopsy	86
4	Fluorescence assays	CRISPR-Cas12a based metal-enhanced fluorescence (MEF) using DNA-functionalized Au nanoparticle (AuNP)	Breast cancer gene-1 (BRCA-1)	Target concentrations ranging from 1 femtomolar to 100 picomolar	30 min	CRISPR Cas12a-assisted AuNP nano sensor without any amplification method	84
		Cas12a activated nuclease poly-T reporter illuminating particles (CANTRIP). CANTRIP consists of CRISPR-Cas12a and TdT-dependent poly(thymine)-templated (copper nanoparticles) CuNPs functional in a single reaction tube	*Bacillus anthracis* lef gene	10 picomolar–2 nanomolar	—	CRISPR-Cas12a and fluorescent CuNPs into a single reaction	87
5	Volumetric bar chart chip	Platinum nanoparticles tethered to magnetic beads by single stranded DNAs for a CRISPR-Cas12a detection	Quantify single nucleotide variations (SNVs)/single nucleotide polymorphisms (SNPs)	Pure DNA samples and mock cell-free DNA samples in serum, with allelic fractions as low as 0.01%	—	This platform enables quantification of multiple cancer mutations in pure DNA samples and mock cell-free DNA samples in serum	83
6	Solid-state nanopore sensors	Solid-state CRISPR-Cas12a-assisted nanopores	HIV-1 DNA	10 nanomolar	60 min	Glass nanopore sensor is effective in monitoring the cleavage activity of the target DNA-activated Cas12a	88
		CRISPR-dCas9 with solid-state nanopores	Detection of targeted DNA	Not reported	60–120 min	The ease of programmability of the gRNA enables versatile detection of a wide range of targets as well as multiplexing	89

## Conclusions and future prospects

Biosensors based on CRISPR/Cas12/13 generate high hopes for the development of potential diagnostics for illness monitoring at an early stage. The majority of the recently introduced CRISPR/Cas biosensing systems have the following advantages: ease of development, ultra-high resolution to single-base change, concentration sensitivity of at least fM or mostly aM, and no requirement for dedicated instrumentation. Because of COVID-19 infection, CRISPR-based diagnostics have picked up steam in the last four years, owing to their specificity, unique *trans*-cleavage, and rapid analysis features, which allow for prompt interventions and interpretation. In recent times, nanotechnology has been combined with CRISPR-based diagnostics to boost system efficiency. Using this technology produces promising results in relevant fields. In the meantime, system stability and applicability should be improved. The introduction of PAM sequences in amplification strategy may overcome PAM dependency. However, these approaches are indirectly dependent on amplification methods. Nowadays, CRISPR diagnostic methods use the *trans*-cleavage activity of CRISPR-Cas to generate amplified detection signals and incorporate CRISPR-Cas after nucleic acid amplification to improve the specificity of assays. Furthermore, additional progress must be made to enhance accuracy since we must be more confident in our trial results before adopting technology universally. At the same time, it will be fascinating to watch the research and development related to the use of CRISPR/Cas in the field of biosensors.

## Data availability

The data sets used and/or analyzed during the current study are available from the corresponding author upon request.

## Conflicts of interest

The authors declare that they have no known competing financial interests or personal relationships that could have appeared to influence the work reported in this paper.
